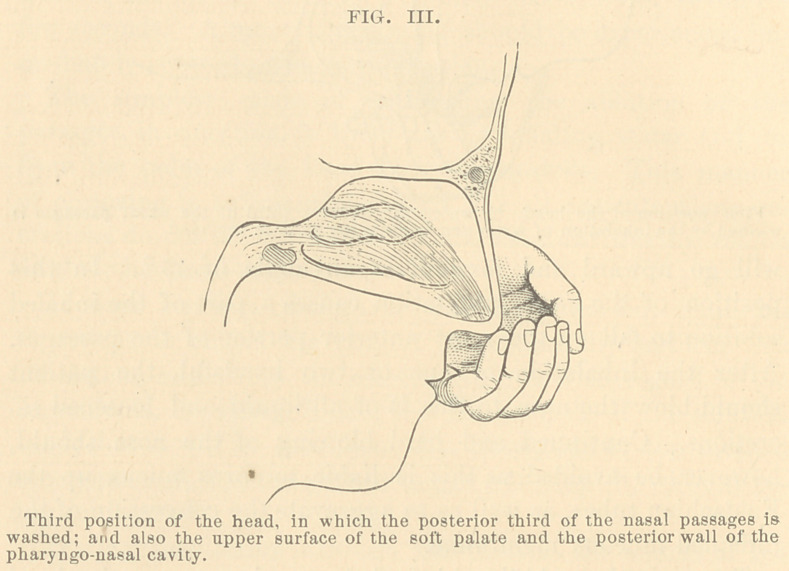# A Simple Mode of Cleansing the Nasal and Pharyngo-Nasal Passages

**Published:** 1877-05

**Authors:** Thomas F. Rumbold

**Affiliations:** 1225 Washington Avenue; St. Louis, Mo.


### EXAMINER.

Vol. XXXIV.—MAY, 1877.—No. 4.

### Original Communications.

### A SIMPLE MODE OF CLEANSING THE NASAL AND PHARYNGO-NASAL PASSAGES.

By THOMAS F. RUMBOLD, M. D., St. Louis, Mo.

The greater number of those patients who are afflicted with chronic catarrhal inflammation of the mucous membrane of the nasal passages, will require, in the early part of their treatment, frequent cleansings of these cavities. As the period between the applications which the physician gives, is frequently so long that the membranes become loaded with accumulated secretion, the patients must attend, in the interim, to the cleansing and to the application of such remedies that can be safely intrusted to their care.

Whenever a collection of muco-purulent secretion is allowed to remain in the nasal or pharyngo-nasal passages, for a length of time, varying from a few hours to a day or more, it becomes fœtid, and acquires an acrid property; this quality is the result of a kind of fermentation, which the heat of the parts causes or favors. If such vitiated secretions are allowed to remain on the mucous membranes, their acridness will aggravate the inflammation. These facts indicate both the necessity of maintaining the catarrhal surfaces in a clean and disinfected condition, and of the patients being instructed as to the most effective mode in which this cleansing may be done.

While it is essential to speedy recovery, to have the nasal passages maintained in a clean condition, it is also equally essential that the means employed in the removal of the secretions should not cause an irritation that will last beyond a few seconds. A sensation of relief should be experienced immediately succeeding each application.

The simplest mode of performing the ablution of the passages, in question, is by means of inhaling water and air from the palm of the hand into the nostrils. This manner of cleansing is sufficiently effective, for all patients whose secretions do not become locked in the nasal cavities by reason of their hardness or size.

It does seem as though it would require but little instructions to enable the patient to successfully perform this inhalation, aside from the directions given with regard to the ingredients, the strength and the temperature of the solution used; but it will be seen from the description of the method recommended, that the patient might not adopt it, without being so directed.

During inspiration through the nostrils, the course of the greatest volume of the stream of air that enters these cavities, is not parallel with the bridge of the nose, nor does it pass along the floor of the nasal passages, but nearly between these two boundaries, which course is generally at an angle of about 45° with the plane of the forehead. If we keep in mind, that the tendency of the stream of inhaled liquid, is to take the same direction that the air does, and that the water, because it is heavier than the air, will deviate from this course by gravitation, we have only to place the head in certain positions, to be enabled to wash or bathe the entire surface of these triangular shaped cavities, except the inferior portions of the turbinated processes.

To reach the anterior third of the nasal cavities, the head of the patient should be inclined forward to such an extent, that the plane of the forehead will be nearly in a horizontal position (Fig. 1); then the stream inhaled from the hand,

FIG. I.

First position of the head, in which the anterior third of the nasal passages is washed by the inhalation of water and air from the palm of the hand.

will go upward and forward at an angle of 45°. In this position of the head, gravitation causes a part of the inhaled solution to fall on the most anterior portion of the passages. After the inhalation of one or two handsful, the patient should blow the nose, to free it of all liquid and loosened secretions. Continued and hard blowing of the nose should, however, be avoided, as this is liable to force mucus up the Eustachian tubes, as well as to aggravate the congestion of the inflamed mucous membrane.

To wash the middle third of the nasal passages, the head should be inclined forward until the forehead is placed at an angle of 45° with the horizon (Fig. 2), then the greater part

FIG. II.

Second position of the head, in which the middle third of the nasal passages is washed by the inhalation of water and air.

of the stream of inhaled air and liquid will enter the cavities in a vertical direction, striking the superior portion of the cavity, but gravitation will divert a part of the fluid forward, and a part of it backward, of the vertical line.

Again the loosened secretions should be blown out.

In the third position of the head, the forehead should be placed in a vertical position (Fig. 3); then the stream of air

FIG. III.

Third position of the head, in which the posterior third of the nasal passages is washed; and also the upper surface of the soft palate and the posterior wall of the pharyngo-nasal cavity.

and fluid will enter the cavities at an angle of 45° with the horizon, going upward and backward. Gravitation, in this case, instead of causing it to fall forward, as it did in the first position, will cause a part of the solution to pass along the floor of the passages, thus washing the remaining third of the surfaces. Again all liquids and loosened secretion should be blown out. In the first and second positions, the inhaled liquid will come out of the nostrils in front; but in the last position, all of it will come out from the mouth.

While the head is in the third position (Fig. 3), it is possible for the patient to inhale the solution with sufficient force, to cause a part of it to strike the posterior wall of the pharyngo-nasal cavity: if so, the surface of this cavity, with that of the pharynx and upper surface of the soft palate, will be washed also. In this way, the patient can remove the tenacious mucus adhering to these surfaces, which removal cannot be accomplished by any other effort he can make, for the reason that the mass of accumulated secretion is located above the place reached by the movements of the tongue, or soft palate, or the force of the breath in hacking or rasping the throat. Patients, in their endeavor to remove this adhering mucus, usually have severe "coughing spells" in the morning, as they turn their efforts to clear the throat, but these efforts do not rid the mucous surface of the offending matter; this removal is accomplished only when they continue to cough long enough to induce gagging efforts, which efforts are accompanied by a qualmish condition of the stomach, and a copious flow of free mucus; it is this fresh flow of liquid mucus that accomplishes the removal of the adhering mass, by washing it away from its place of lodgment. The attempt to remove this tenacious secretion by the old gargling method, must always fail, because this method cannot throw the liquid, employed, to the location desired; it can only wash the tonsils, the anterior surface of the soft palate, the base of the tongue, and a small unimportant portion of the fauces.

Those patients who cannot clear their throat with the first course of inhalation from the hand, and whose cough is continued so long, by the presence of the lodged secretion, that it produces a gagging sensation, should lie down in bed for a few minutes, as the recumbent position will usually relieve this disagreeable symptom. After the sickness of the stomach has passed off, and the solution inhaled has loosened the adhering mass, they will be enabled to clear the throat by another course of inhalation.

During the last eleven years, I have recommended this method to my patients, they have found that it had a very beneficial effect, always freeing the nasal and pharyngo-nasal passages of the accumulated secretions.

The number of times that these inhaling operations should be repeated, is a matter of some importance. We must keep in mind that the nasal passages are not made to receive any kind of liquid, and that the lining membranes absorb, to their injury, more or less of every fluid that comes in contact with them. The reason why the medicated solution is a benefit, is, because it acts as a solvent to vitiated secretions that are far more deleterious to the mucous membranes, than the effect of the absorption of the liquid itself: it follows, therefore, that just so soon as the decomposed secretions are removed, the solution, if continued, will be a means of doing harm. In other words, the washing out of these cavities is but a choice between two evils, the use of the solution being the lesser. It is evident, then, that the sooner the lesser evil is discontinued, after the greater evil has been removed, the better it will be for the mucous membranes.

After the surfaces have been made clean, the washing should be stopped, even though it produces a pleasing sensation, because the absorption of the water causes the membranes to become swollen, in which condition they are more susceptible to the deleterious influences of cold.

If at any time the inhaled liquid produces a painful sensation, which lasts beyond one or two seconds, then it should be discontinued, even if the passages are not entirely cleansed. With such cases, a few partial washings, aided by the local applications, made by the physician, will decrease the heat of the parts, that is the cause of the hardening of the secretions; then the cleansing can be completed without producing the least disagreeable effect.

Patients in whose nostrils or throat dry masses collect, should inhale three handsful of the solution immediately on getting out of bed in the morning, placing the head in the three positions named; this will soften the mass a little; by the time they have completed their toilet, they will probably be able to cleanse the head by a second course, *i. e.*, with three handsful more. During the early treatment of a bad case, three and four courses may be required in the forenoon.

The solution to be inhaled from the hand is composed of common table salt and water, that is a little warmer than blood heat, about one teaspoonful of the former to a pint of the latter. Patients will soon learn, from experience, whether or not this is the proper strength or temperature, when they are informed that water, either without salt, or with too much in it, is productive of more or less pain, and that the right quantity (which varies with different individuals) produces a pleasant bland sensation; also, that cold water causes a disagreeable as well as an injurious effect.

For those cases whose nasal secretions are offensive, five grains of salicylic acid should be added to the pint of warm salt water.

1225 Washington Avenue.

## Figures and Tables

**FIG. I. f1:**
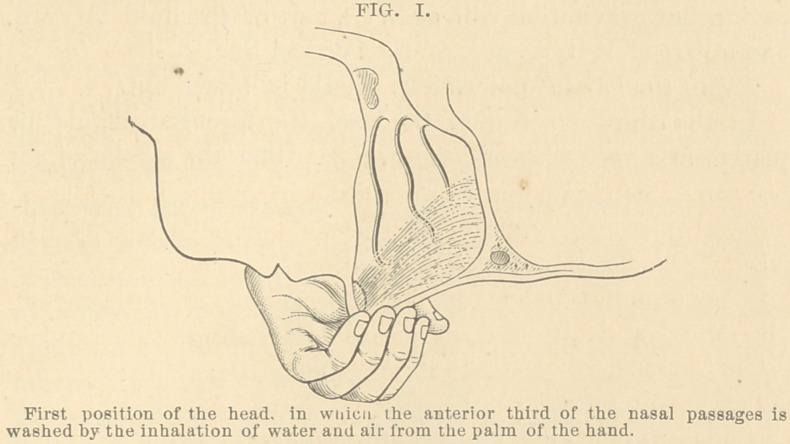


**FIG. II. f2:**
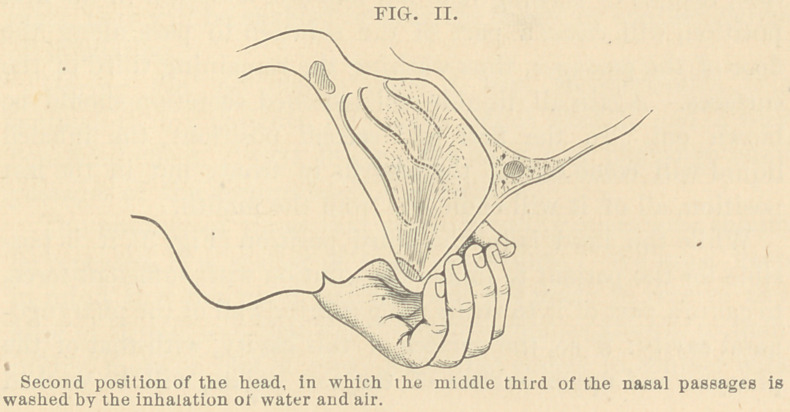


**FIG. III. f3:**